# Endoscopic dilation of benign esophageal anastomotic strictures over 16 mm has a longer lasting effect

**DOI:** 10.1007/s00464-016-5187-0

**Published:** 2016-09-01

**Authors:** Emo E. van Halsema, Irma C. Noordzij, Mark I. van Berge Henegouwen, Paul Fockens, Jacques J. Bergman, Jeanin E. van Hooft

**Affiliations:** 1 Department of Gastroenterology and Hepatology, C2-116, Academic Medical Center, Meibergdreef 9, 1105 AZ Amsterdam, The Netherlands; 2 Department of Surgery, Academic Medical Center, Amsterdam, The Netherlands

**Keywords:** Endoscopic dilation, Surgical anastomosis, Esophagectomy, Benign esophageal strictures, Esophageal stenosis, Esophagoscopy

## Abstract

**Background:**

The optimal target of endoscopic dilation of postsurgical esophageal strictures is unknown. Our aim was to compare the dilation-free period of patients who underwent dilation up to 16 mm with patients who were dilated up to 17 or 18 mm.

**Methods:**

We retrospectively analyzed adult patients who received bougie/balloon dilation for a benign anastomotic stricture after esophagectomy. An anastomotic stricture was defined as dysphagia in combination with a luminal diameter of ≤13 mm at endoscopy. We analyzed the dilation-free period using Kaplan–Meier and multivariable Cox regression analysis.

**Results:**

Eighty-eight patients were dilated up to a maximum diameter of 16 mm and 91 patients to a diameter >16 mm. The stricture recurrence rate was 79.5 % in the 16 mm group and 68.1 % in the >16 mm group (*p* = 0.083). The overall dilation-free period had a median of 41.5 (range 8–3233) days and 92 (range 17–1745) days, respectively (*p* < 0.001). For patients who developed a stricture recurrence, the median dilation-free period was 28 (range 8–487) days and 63 (range 17–1013) days, respectively (*p* = 0.001). Cox regression analysis showed a reduced risk of stricture recurrence for patients who were dilated up to >16 mm: crude hazard ratio (HR) 0.57 (95 % confidence interval (CI) 0.41–0.81) and adjusted HR 0.48 (95 % CI 0.33–0.70).

**Conclusions:**

Endoscopic dilation over 16 mm resulted in a significant prolongation of the dilation-free period in comparison with dilation up to 16 mm in patients with benign anastomotic strictures after esophagectomy.

**Electronic supplementary material:**

The online version of this article (doi:10.1007/s00464-016-5187-0) contains supplementary material, which is available to authorized users.

Esophageal cancer is globally among the top ten of cancers with the highest incidence and death rates [1]. It was estimated that in the US in the year 2015 almost 17.000 new cases of esophageal cancer would be diagnosed and almost 15.600 deaths attributable to esophageal cancer would occur [2]. Potentially curable esophageal cancer is generally treated with a multimodal approach that includes surgery [3]. After esophagectomy a cervical or intrathoracic anastomosis is constructed using a gastric tube, or in rare cases a colonic or jejunal interposition, for esophageal replacement.

One of the major complications after esophagectomy is the development of benign anastomotic strictures, which occurs in 10–43 % of patients [4–8]. Esophageal strictures cause complaints of dysphagia and weight loss, are associated with a decreased quality of life and lead to additional health care costs [9]. Endoscopic bougie or balloon dilation is currently the standard treatment to resolve dysphagia caused by benign esophageal strictures [10]. To prevent dilation-related adverse events, the ‘rule of three’ is usually applied with the use of bougie dilators. This means that the stricture is dilated no more than three millimeters using three consecutive bougies once moderate resistance is encountered [11]. Patients hereby require repeated endoscopies to reach a satisfactory luminal diameter. When a luminal diameter of 13–15 mm has been reached, patients are able to tolerate a normal diet [11, 12]. However, approximately 50 % of patients with benign esophageal anastomotic strictures develop recurrent complaints of dysphagia, requiring again repeated dilation procedures [13, 14]. Besides this major burden for patients, the repeated endoscopic dilations consequently impact on the costs of healthcare and contribute to sickness absence of patients as well.

The optimal target diameter of endoscopic dilation of benign esophageal strictures is unknown and therefore an arbitrary measure. To ensure luminal patency, patients are usually dilated to 16–20 mm [13–16]. It is unknown whether a larger target diameter increases the risk of perforation. So far, no studies have found a correlation between the size of the balloon or bougie dilator and the occurrence of perforation [17, 18]. Increasing the target diameter will result in additional endoscopic procedures, especially when the ‘rule of three’ is applied with the use of bougie dilators. The question is whether those last additional millimeters past the 16 mm are effective. Therefore, our aim was to compare the dilation-free period for patients who underwent endoscopic dilation up to 16 mm with patients who were dilated to more than 16 mm.

## Materials and methods

This study was designed as a retrospective, single center, cohort study and was approved by the Medical Ethics Committee of the Academic Medical Center, Amsterdam, the Netherlands. We performed an electronic search through our endoscopic database ENDOBASE (Olympus Medical Systems, Hamburg, Germany) to identify patients who underwent upper gastrointestinal endoscopy and dilation therapy between January 2005 and June 2015. In attempt to select a large and homogeneous population, we included adult patients who received bougie or balloon dilation for a benign anastomotic stricture after esophagectomy with either gastric tube reconstruction or colonic interposition for esophageal replacement. Patients were excluded if they had active esophageal malignancy, strictures other than anastomotic strictures, esophagectomy before January 2000, esophagectomy or endoscopic dilation outside our institution, other endoscopic therapies including stent placement or incision therapy, persisting postsurgical esophageal fistula at time of first dilation, or dilation-related perforation or fistula. We applied the following definition for a benign anastomotic stricture: dysphagia in combination with a luminal diameter ≤13 mm at the site of the anastomotic stricture diagnosed at endoscopy. The luminal diameter was based on the report of resistance during the passage of a ≤13 mm bougie or the inability to pass the stricture with a diagnostic or therapeutic gastroscope. We divided patients into two groups based on the maximum luminal diameter that was reached with endoscopic dilation during the last endoscopy of the initial treatment: (1) 16 mm and (2) >16 mm. The primary outcome of the study was the dilation-free period, defined as the period between the date of reaching the maximum diameter at endoscopic dilation and the date of endoscopic re-intervention for stricture recurrence or end of follow-up without the need for additional endoscopic dilation. Secondary outcomes were the stricture recurrence rate and serious dilation-related adverse events. We defined stricture recurrence as dysphagia requiring endoscopic dilation in the absence of locoregional tumor recurrence. Follow-up ended when patients developed a recurrent stricture, metastatic disease or local tumor recurrence, or at last contact. The patients who developed a recurrent stricture and underwent endoscopic re-intervention with bougie or balloon dilation were analyzed as well for the dilation-free period after endoscopic re-intervention. For this purpose, we also divided these patients into the groups (1) 16 mm reached after endoscopic re-intervention and (2) >16 mm reached after endoscopic re-intervention.

### Data collection

We retrospectively collected the baseline variables from the medical records that are presented in Table 1. The following variables were also included in the data collection: the number of endoscopies needed to reach the target diameter; maximum luminal diameter reached; date of reaching the target diameter; endoscopic re-intervention for stricture recurrence; date of endoscopic re-intervention; date of last dilation-free follow-up; and serious dilation-related adverse events. We also collected the equivalent variables when patients underwent endoscopic re-intervention dilation for a recurrent stricture. The stricture diameter was based on the size of the first bougie that was passed with resistance. In the few cases that these data were not available, the stricture diameter was estimated by EvH, IN and JvH in a consensus meeting using the endoscopic images of the untreated stricture. To determine the location of the stricture, the esophagus was divided into three segments: proximal (<25 cm from the incisors), mid (25–30 cm from the incisors) and distal (>30 cm from the incisors).

**Table 1:** Baseline characteristics

	16 mm *N* = 88	>16 mm *N* = 91	*p*
Gender			0.606
Male	64 (72.7)	63 (69.2)	
Female	24 (27.3)	28 (30.8)	
Age (years); mean ± SD	64.3 ± 8.2	63.3 ± 10.6	0.487
Esophageal replacement			0.240
Gastric tube reconstruction	86 (97.7)	91 (100)	
Colonic interposition	2 (2.3)	0 (0)	
Location of esophageal anastomosis			0.182
Cervical	77 (87.5)	86 (94.5)	
Intrathoracic	10 (11.4)	5 (5.5)	
*Missing*	1 (1.1)	0 (0)	
Esophageal anastomosis			0.193
Hand-sewn	60 (68.2)	58 (63.7)	
Stapled	12 (13.6)	5 (5.5)	
*Missing*	16 (18.2)	28 (30.8)	
Esophageal anastomosis^a^			**0.024**
End-to-end	40 (45.5)	53 (58.2)	
End-to-side	37 (42.0)	23 (25.3)	
*Missing*	11 (12.5)	15 (16.5)	
Postsurgical esophageal leakage			0.083
Yes	18 (20.5)	29 (31.9)	
No	70 (79.5)	62 (68.1)	
Stent for postsurgical leakage			1.000
Yes	1 (1.1)	1 (1.1)	
No	87 (98.9)	90 (98.9)	
Days between surgery and first dilation; median (range)	66 (31–399)	77 (28–680)	0.255
Stricture diameter (mm); mean ± SD	9.8 ± 1.9	9.5 ± 1.9	0.305
Esophageal segment			**0.013**
Proximal (<25 cm from incisors)	74 (84.1)	87 (95.6)	
Mid (25–30 cm from incisors)	14 (15.9)	4 (4.4)	
Distal (>30 cm from incisors)	0 (0)	0 (0)	
Method of endoscopic dilation			1.000
Bougie	85 (96.6)	87 (95.6)	
Balloon	0 (0)	1 (1.1)	
Combination	3 (3.4)	3 (3.3)	
Kenacort injected during dilation^b^			**0.001**
Yes	9 (10.2)	0 (0)	
No	79 (89.8)	91 (100)	

^a^One patient with a side-to-side anastomosis was added to the end-to-side group

^b^Patients who received Kenacort participated in the trial by Hirdes et al. [16] *SD* standard deviation

### Procedures

Although the procedures in this study were not standardized because of the retrospective nature of this study, we give a description of how endoscopic dilation was usually performed according to our local protocol. Endoscopic dilation was performed as an outpatient procedure. Patients were asked to fast for at least 6 h before the procedure. Anticoagulants were stopped 4–6 days before the procedure. Monotherapy of a prophylactic dose of an antiplatelet drug could be continued. No prophylactic antibiotics were administered. Patients received either ‘deep sedation’ using propofol under the supervision of an anesthesia team or ‘conscious sedation’ using midazolam and/or fentanyl administered by the endoscopist. Because bougie dilators are equally effective in comparison with balloons [19–21], but less expensive because of their re-usability, we preferred dilation with the use of Savary-Gilliard bougies and fluoroscopic guidance. Depending whether a diagnostic gastroscope (diameter < 10 mm) could pass or not pass the stricture, we started with an 11 mm or 8 mm bougie, respectively. Patients were dilated up to a satisfactory luminal diameter based on the discretion of the endoscopist using the ‘rule of three’, which means that the stricture was dilated no more than three millimeters per procedure once resistance had been encountered. Patients were discharged 1–2 h after the intervention after drinking a glass of water under the supervision of the endoscopist. Consecutive dilation procedures were scheduled within 1–2 weeks until a target diameter of at least 16 mm was reached. The vast majority of procedures were performed or supervised by six endoscopists dedicated to interventional endoscopy. The final target diameter was an arbitrary measure that mainly depended on the preference of the endoscopist performing the procedure. The patient was then discharged and instructed to contact the outpatient clinic in case of recurrent dysphagia.

### Statistical analysis

We described continuous variables as mean with standard deviation (SD) and median with lowest and highest value (range) when they had a normal and skewed distribution, respectively. For the comparison of variables with a normal and skewed distribution, the independent sample *t* test and the Mann–Whitney U test were used, respectively. We used Pearson’s Chi-square or Fisher’s exact test, depending on the number of cases, to compare categorical variables. Besides the Mann–Whitney U test, a Kaplan–Meier analysis with log-rank test was performed for the comparison of the dilation-free period between dilation up to 16 and >16 mm. We analyzed time to stricture recurrence using a multivariable Cox proportional hazards regression model, including the variables with *p* < 0.1 in univariable analysis. No critical violations of the proportional hazards assumption were found using the log minus log plot of each variable included in the multivariable model. We handled missing data as ‘missing completely at random’ and therefore performed a complete case analysis (*n* = 153; missing *n* = 26). Two-sided p values <0.05 were considered statistically significant. We used the statistical software SPSS Statistics version 22 (IBM corp., Armonk, New York, USA) for the analyses.

## Results

**Fig. 1:**
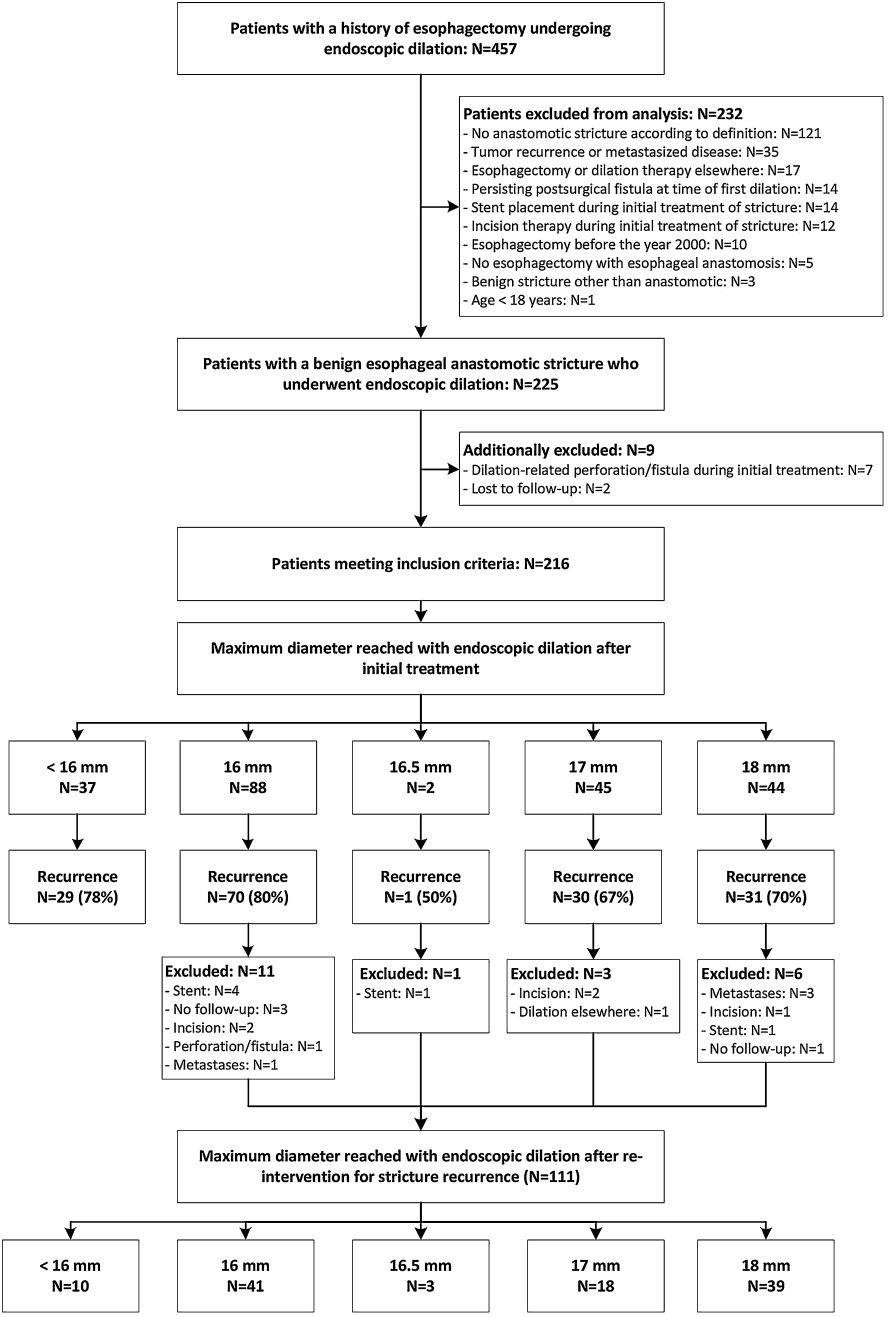
Flow chart of the study

**Fig. 2:**
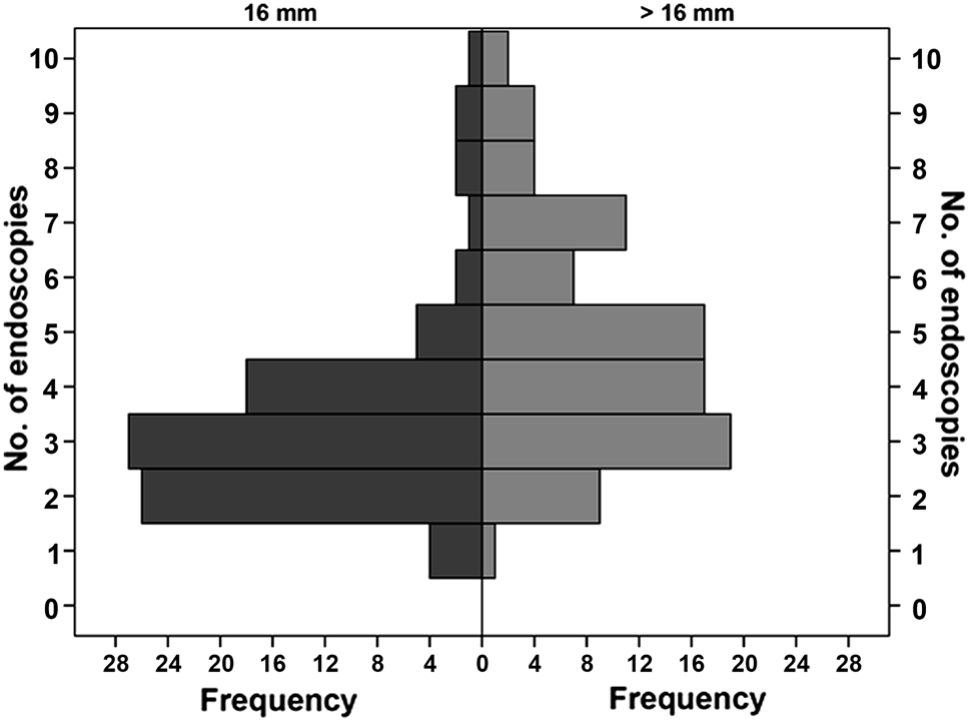
Number of endoscopies needed to reach the maximum target diameter

Between January 2005 and June 2015, we identified 457 patients who underwent endoscopic dilation at the Academic Medical Center after esophageal surgery, of whom 225 patients fulfilled the inclusion criteria (Fig. 1). The patients who developed a dilation-related perforation or fistula during the initial treatment (3.1 %; 7/225) and who had no follow-up data available (0.9 %; 2/225) were additionally excluded. Of the remaining 216 patients, 179 reached a maximum target diameter of at least 16 mm after endoscopic dilation and were included in the final analysis (Fig. 1). Eighty-eight patients were dilated up to 16 mm and 91 patients up to a diameter of >16 mm, including 16.5 mm (*n* = 2), 17 mm (*n* = 45) and 18 mm (*n* = 44). The baseline characteristics of the two groups are presented in Table 1. The median number of endoscopies needed to reach the maximum target diameter was 3 (range 1–10) and 4 (range 1–10) in the 16 and >16 mm group, respectively (*p* < 0.001; Fig. 2). The period from the first dilation to reaching the maximum diameter had a median of 15 (range 0–82) days in the 16 mm group and 25 (range 0–85) days in the >16 mm group (*p* < 0.001).

**Fig. 3:**
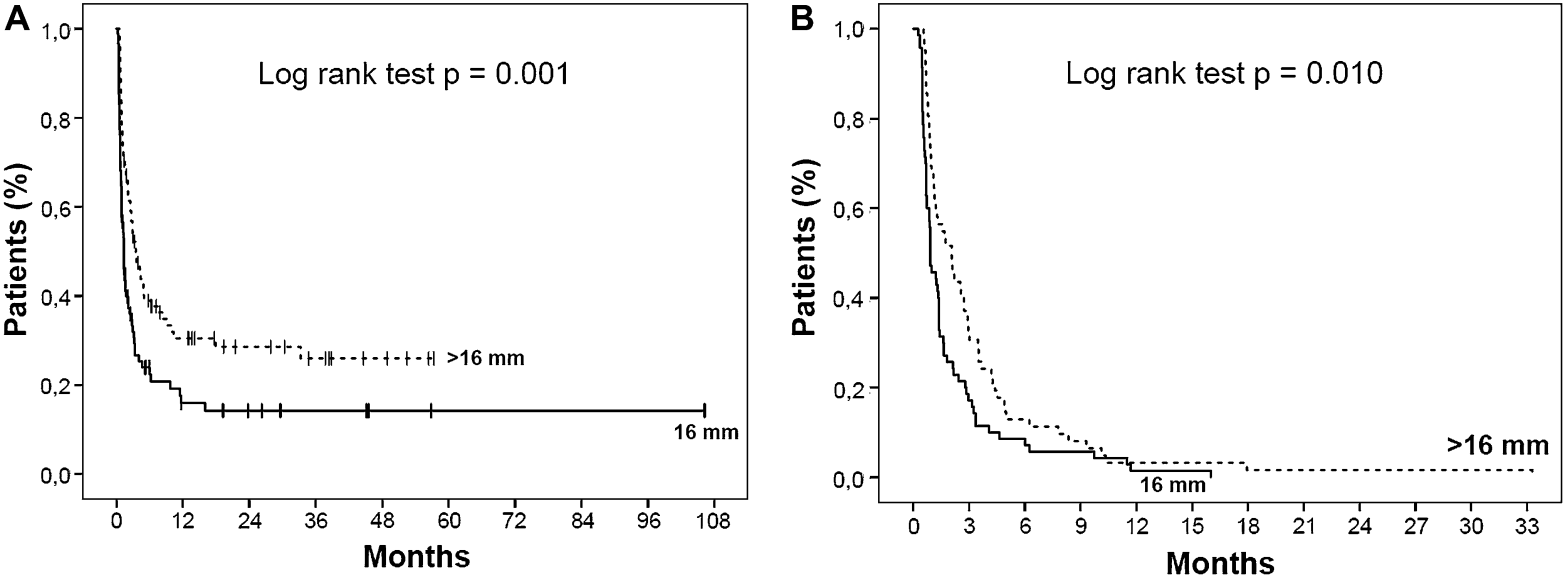
**A** Dilation-free period in all patients undergoing endoscopic dilation to a maximum diameter of 16 mm (*N* = 88) and >16 mm (*N* = 91). **B** Dilation-free period for those patients who developed a recurrent stricture after endoscopic dilation to a maximum diameter of 16 mm (*N* = 70) and >16 mm (*N* = 62)

The stricture recurrence rate was 79.5 % in the 16 mm group and 68.1 % in the >16 mm group (*p* = 0.083). The overall dilation-free period had a median of 41.5 (range 8–3233) days in the 16 mm group and 92 (range 17–1745) days in the >16 mm group (*p* < 0.001). For patients who developed a stricture recurrence, the median dilation-free period was 28 (range 8–487) days and 63 (range 17–1013) days, respectively (*p* = 0.001). Kaplan–Meier analyses with log-rank test are presented in Fig. 3A, B. Cox regression analysis showed a reduced risk of stricture recurrence for patients who were dilated to >16 mm in comparison with the 16 mm group: crude hazard ratio (HR) 0.57 (95 % confidence interval (CI) 0.41–0.81) and adjusted HR 0.48 (95 % CI 0.33–0.70). Details are presented in Table 2.

**Table 2:** Cox regression analysis of factors associated with time until stricture recurrence

	HR (95 % CI)		*p*	
	Crude	Adjusted	Crude	Adj.
Anastomosis (end-to-end); *missing N* = *26*	1.16 (0.80–1.70)	1.42 (0.95–2.13)	0.434	0.087
Postsurgical leakage (yes)	1.07 (0.73–1.57)	1.01 (0.67–1.51)	0.731	0.983
Esophageal segment (proximal)	0.83 (0.48–1.45)	0.52 (0.24–1.16)	0.512	0.110
Kenacort injected (*yes*)	1.32 (0.61–2.82)	0.96 (0.41–2.28)	0.481	0.928
Maximum diameter reached (>16 mm)	0.57 (0.41–0.81)	0.48 (0.33–0.70)	0.001	0.000

*NB* Complete case analysis includes 153 patients (26 missing), *HR* hazard ratio, *CI* confidence interval; *adj.* adjusted

### Subgroup analysis

**Fig. 4:**
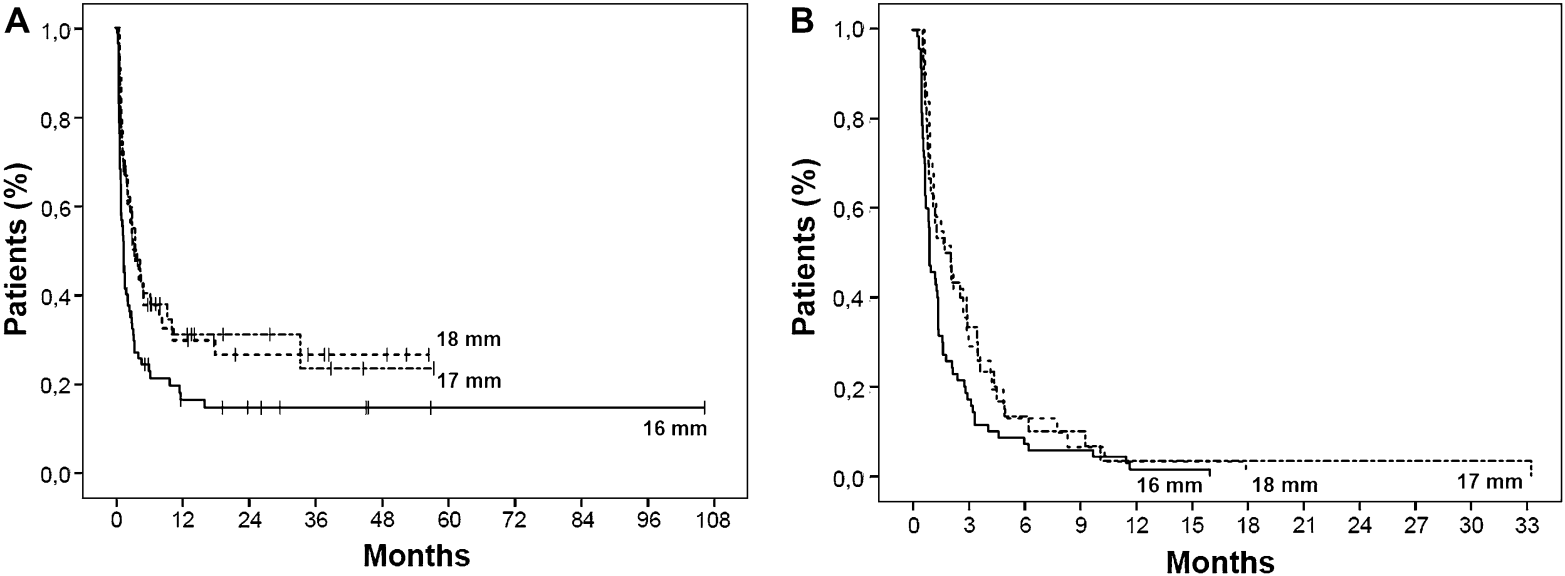
**A** Dilation-free period in all patients undergoing endoscopic dilation to a maximum diameter of 16 mm (*N* = 88), 17 mm (*N* = 45) and 18 mm (*N* = 44). **B** Dilation-free period for those patients who developed a recurrent stricture after endoscopic dilation to a maximum diameter of 16 mm (*N* = 70), 17 mm (*N* = 30) and 18 mm (*N* = 31)

To study the effect of each millimeter increase in maximum diameter in comparison with the 16 mm group, we divided the patients into three groups based on the maximum diameter reached after endoscopic dilation: 16 mm (*n* = 88), 17 mm (*n* = 45) and 18 mm (*n* = 44). The stricture recurrence rates were 79.5, 66.7 and 70.5 %, respectively (*p* = 0.228). Analyzed separately, dilation up to 17 mm and dilation up to 18 mm significantly (*p* < 0.01) increased the dilation-free period in comparison with the 16 mm group (Table 3; Fig. 4A, B).

**Table 3:** Time to stricture recurrence

	16 mm	17 mm	18 mm
Overall dilation-free period^a^			
Median (days)	41.5	106	91
R ange (days)	8–3233	17–1745	20–1718
*p* value	*Reference*	0.003	0.001
Time to stricture recurrence^b^			
Median (days)	28	58	63
Range (days)	8–487	17–1013	20–546
*p* value	*Reference*	0.018	0.003

^a^Until stricture recurrence or end of follow-up

^b^Includes only the patients who developed a recurrent stricture

### Endoscopic re-intervention for stricture recurrence

**Fig. 5:**
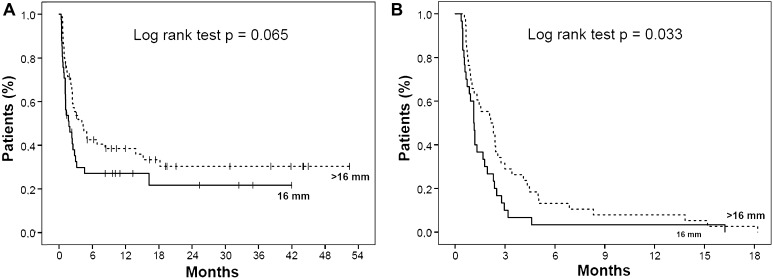
**A** Dilation-free period in all patients undergoing endoscopic re-intervention dilation for a recurrent stricture to a maximum diameter of 16 mm (*N* = 41) and >16 mm (*N* = 60). **B** Dilation-free period for those patients who developed a second stricture recurrence after endoscopic re-intervention dilation to a maximum diameter of 16 mm (*N* = 30) and >16 mm (*N* = 38)

After the initial treatment 132 patients (73.7 %; 132/179) developed a recurrent stricture, of whom 116 patients fulfilled the inclusion criteria again (Fig. 1). The patients who developed a dilation-related perforation or fistula during re-intervention (0.9 %; 1/116) and who had no follow-up data available (3.4 %; 4/116) were additionally excluded. Of the remaining 111 patients, 101 reached a maximum target diameter of at least 16 mm after endoscopic dilation and were included in the re-intervention analysis (Fig. 1). Forty-one patients were dilated up to 16 mm and 60 patients up to a diameter of >16 mm, including 16.5 mm (*n* = 3), 17 mm (*n* = 18) and 18 mm (*n* = 39). The mean diameter of the untreated recurrent stricture was similar between the 16 mm and >16 mm group: 12.4 (SD 1.7) mm and 12.7 (SD 2.9) mm (*p* = 0.501). After re-intervention, the stricture recurrence rate was 73.2 % in the 16 mm group and 63.3 % in the >16 mm group (*p* = 0.301). The overall dilation-free period had a median of 51 (range 11–1277) days in the 16 mm group and 93 (range 17–1596) days in the >16 mm group (*p* = 0.024). For patients who developed a second stricture recurrence, the median dilation-free period after re-intervention was 34.5 (range 11–494) days and 67.5 (range 17–554) days, respectively (*p* = 0.025). Kaplan–Meier analyses with log-rank test are presented in Fig. 5A, B.

### Adverse events

Out of the 225 patients and the 1309 endoscopic dilation procedures that we analyzed, 13 dilation-related adverse events were reported in 12 patients, making the adverse event rate 5.3 % per patient and 1.0 % per procedure. Two patients developed three episodes of postprocedural bleeding that required endoscopic treatment with adrenaline in one case and endoscopic inspection without intervention in the remaining two cases. Two patients had a large ulcer at the anastomosis, discovered during the next scheduled dilation procedure, which resulted in postponement of further dilation. Eight patients (3.6 % per patient and 0.6 % per procedure) developed a perforation or fistula following endoscopic bougie dilation. The details of these cases are described in Table 4. One patient, of whom no follow-up data were available, died of an unknown cause three days after an endoscopic dilation procedure. Two adverse events, a large ulcer and a perforation, developed after dilation up to 17 mm and 18 mm, respectively. The remaining adverse events all occurred after dilation up to 16 mm or less.

**Table 4:** Dilation-related perforation or fistula

Sex, age	Stricture diameter (mm)	No. of previous dilations	Diameter reached (mm)	No. of days postsurgery	Outcome
M, 75y	7	1	9	92	Endoscopic treatment with Sumptube and pleural drainage of empyema. Refractory stricture with persisting fistula, causing recurrent pneumonias. Received a partially covered metal stent 10 months later to seal off the fistula. Died because of tumor recurrence with stent in situ
M, 76y	6	3	13	69	Contrast study during endoscopy shows leakage toward the respiratory tract. Recovered within 2 weeks after conservative treatment with antibiotics
M, 77y	8	1	8	118	Fausse route with 8 mm bougie. Recovered after conservative treatment with partially covered metal stent and antibiotics
M, 63y	9	4	15	59	Respiratory fistula. Received airway stent followed by esophageal stent because of persisting leakage. Passed away one month later
F, 71y	11^a^	5	13	71	Patient developed respiratory complaints and pneumonia following dilation. Recovered after conservative treatment with antibiotics
F, 63y	8	4	15	64	Respiratory fistula. Recovered after a long period (<1 year) of conservative treatment
M, 66y	7	9	18	103	Perforation conservatively treated with Sumptube for suction. Recovered within 1 month
M, 49y	7	1	9	66	Suspicion of dehiscence of anastomosis with two small fistulas. Recovered within 2 months after conservative treatment

^a^Stricture diameter at recurrence. *M* male, *F* female, *y* years

## Discussion

In this study we demonstrated that endoscopic dilation of benign esophageal anastomotic strictures to a target diameter of more than 16 mm was associated with a statistically significant prolongation of the dilation-free period in comparison with dilation to a target diameter of 16 mm. This finding was valid for the initial treatment of newly diagnosed anastomotic strictures, as well as for the re-intervention dilation of recurrent strictures. Dilation to >16 mm also resulted in an 11.4 % decrease of the stricture recurrence rate after the initial treatment. Although this decrease was not statistically significant, it showed a trend toward significance (*p* = 0.083). These results have led to a change in our management and benign esophageal anastomotic strictures are now always dilated to a target diameter of 18 mm at our institution.

To our knowledge, there are no studies that investigated the optimal target diameter of endoscopic dilation. To relieve dysphagia, guidelines recommend to dilate up to 13–15 mm [11, 22]. However, since benign esophageal strictures tend to recur frequently, it is much more common to dilate to at least 16 mm and even up to 18 or 20 mm, especially in patients with anastomotic and peptic strictures [13–15, 17, 19, 21, 23–25]. A retrospective study that included patients with esophagojejunal anastomotic strictures after total gastrectomy, compared three groups of patients based on the maximum diameter of balloon dilation and number of endoscopic sessions [26]. Unfortunately, the sample size and number of events were insufficient to draw valid conclusions. Another retrospective study, including 155 patients who underwent balloon dilation for anastomotic strictures after esophagectomy, found no correlation between a balloon size of less than 20 mm and the risk of stricture recurrence (odds ratio 1.74; 95 % CI 0.89–3.39) [13]. In 89 % of patients, a maximum balloon size of 20 mm was used, resulting in a stricture recurrence rate of 50 % [13]. This was much lower than the recurrence rate of 74 % (132/179) in our cohort, in which almost 50 % of patients were dilated to a maximum target diameter of only 16 mm. Although the definition of an anastomotic stricture was not clearly defined in the aforementioned study [13], these results also suggest that dilation to >16 mm is more effective.

Although we found a clinically relevant prolongation of the dilation-free period after dilation to >16 mm, one might question the cost-effectiveness of increasing the target diameter to 17 or 18 mm. In our study, patients had a 1.1–1.6 months prolongation of their dilation-free period with a median increase of one endoscopy and a mean increase of 1.41 endoscopies. We think that the benefits of dilation to >16 mm are worth the extra effort. The costs of the extra endoscopy may finally even out, because patients who are dilated to 16 mm return sooner with recurrent dysphagia and their strictures tend to recur more frequently. Since patients are treated more effectively with dilation to >16 mm, the extra endoscopy may even reduce sickness absence and contribute to labor participation.

Besides a potential benefit in cost-effectiveness, more effective treatment of benign esophageal anastomotic strictures may also affect the quality of life. A prospective cohort study demonstrated that patients who developed an anastomotic stricture after esophageal resection had significantly poorer scores of global quality of life and social function at six months after discharge in comparison with patients without anastomotic strictures [9]. So dilation up to >16 mm may result in a more rapid recovery of the quality of life to a level that is comparable with patients without anastomotic strictures.

Another important question is whether dilation to >16 mm is safe and does not increase the risk of esophageal perforation. In our study one out of the eight perforations occurred after dilation to >16 mm, which demonstrates that the target diameter of endoscopic dilation for benign esophageal anastomotic strictures can be safely increased up to 17 or 18 mm. The overall perforation rate of 0.6 % per procedure in our series is comparable to the perforation rates reported in the literature after endoscopic dilation of benign esophageal anastomotic strictures, which varied from 0–1.8 % [12, 13, 16–18, 23, 24, 27, 28]. A retrospective series that focused on the incidence and management of esophageal ruptures after endoscopic balloon dilation of benign esophageal strictures, reported a perforation rate of 1.8 % (13/736) per procedure in patients with postoperative strictures [17]. However, these perforations mainly included type two ruptures (12/13) with contrast leakage restricted to the immediately adjacent area that could be managed conservatively [17]. In this study and another large series on balloon dilation for postesophagectomy strictures, no correlation was found between a larger balloon size and the occurrence of esophageal ruptures [13, 17].

One might also question whether the ‘rule of three’ with the use of bougies is really necessary and whether strictures can also safely be dilated four or five millimeters per session. This would result in a reduction of the number of endoscopies needed to reach the maximum target diameter. In a retrospective study on the incidence and outcomes of bougienage for anastomotic strictures, the authors suggested that ‘the extent of the first bougienage should not depend on a rigid rule but on careful evaluation consistent with the anastomotic stricture’ [15]. Several studies using balloon dilation already showed that dilation over 3 mm per session was safe and feasible. For instance, Park et al. reported that 89 % of patients with anastomotic strictures were dilated with a maximum balloon size of 20 mm during the initial dilation session with no major complications after the first session [13]. In another retrospective study, balloon sizes of 12–15 and 15–18 mm diameter were used in patients with severe (<5 mm diameter) and moderate (5–10 mm diameter) strictures, respectively [24]. Patients were asked to ring a bell if they experienced discomfort during the balloon dilation. Using this strategy, the authors reported a perforation rate of 0.3 % per procedure [24]. A retrospective study that included patients with esophagojejunal anastomotic strictures of a median diameter of 5 or 6 mm, also reported that 66 % (38/58) of patients could be dilated up to 16.5–20 mm in one or two sessions, which resulted in a single perforation [26]. However, the cost-effectiveness of dilation to >16 mm and the maximum increase in diameter during a single session of endoscopic dilation might be the subject of future research.

The main limitation of our study is the nonrandomized, retrospective design. To achieve a valid comparison between dilation to >16 mm with dilation up to 16 mm, we collected the variables that have been identified in the literature to correlate with stricture development or stricture severity and adjusted for the variables that were unequally distributed in a multivariable Cox regression analysis. Because we are dealing with selected populations, one might question whether the patients who only reached 16 mm after endoscopic dilation did not have severer and tighter strictures than the patients who reached >16 mm. However, the equal stricture diameter at baseline and the significantly fewer endoscopic sessions in the 16 mm group (Fig. 2) plead against this assumption. Furthermore, because of the retrospective design the adverse event rate is most likely underestimated due to underreporting.

In conclusion, increasing the target diameter of endoscopic dilation up to 17 or 18 mm is safe and feasible in benign anastomotic strictures after esophagectomy. Dilation over 16 mm resulted in a significant prolongation of the dilation-free period in comparison with dilation up to 16 mm. Therefore, increasing the target diameter of endoscopic dilation up to 17 or 18 mm is more effective.

## Electronic supplementary material

Below is the link to the electronic supplementary material.

Supplementary material 1 (DOC 31 kb)
