# Supplementary material for: Endoscopic dilation of benign esophageal anastomotic strictures over 16 mm has a longer lasting effect
Source: Surg Endosc. 2016 Sep 1;31(4):1871–81. doi: 10.1007/s00464-016-5187-0 (PMC5346152; doi:10.1007/s00464-016-5187-0)
Supplement: Supplementary file 1 — Supplementary material 1 (DOC 31 kb) [file 464_2016_5187_MOESM1_ESM.doc]

**Supplementary file**

Table 5. Dilation groups categorized by location of the anastomosis and type of anastomosis

|  | **16 mm**  **(n=88)** | **> 16 mm**  **(n=91)** |
| --- | --- | --- |
| **Cervical anastomosis** | 77 (87.5) | 86 (94.5) |
| End-to-end  End-to-side  Missing | 38 (43.2)  36 (40.9)  3 (3.4) | 52 (57.1)  21 (23.1)  13 (14.3) |
| Hand-sewn  Stapled  Missing | 58 (65.9)  4 (4.5)  15 (17.0) | 57 (62.6)  1 (1.1)  28 (30.8) |
| **Intrathoracic anastomosis** | 10 (11.4) | 5 (5.5) |
| End-to-end  End-to-side  Missing | 2 (2.3)  1 (1.1)  7 (8.0) | 1 (1.1)  2 (2.2)  2 (2.2) |
| Hand-sewn  Stapled  Missing | 2 (2.3)  8 (9.1)  0 (0) | 1 (1.1)  4 (4.4)  0 (0) |

NB. in one patient in the 16 mm group the location of the anastomosis (cervical/intrathoracic) was unknown
